# Channelopathy linking *KCNH2* mutation and primary aldosteronism: a case of life-threatening torsades de pointes

**DOI:** 10.1210/jcemcr/luag123

**Published:** 2026-04-28

**Authors:** Saran Dejprapasorn, Sunti Limumpornpetch, Watchara Lohawijarn, Pongsakorn Choochuen, Wongsakorn Chaochankit, Padiporn Limumpornpetch

**Affiliations:** Endocrinology and Metabolism Unit, Division of Internal Medicine, Faculty of Medicine, Prince of Songkla University, Hat Yai, Songkhla 90110, Thailand; Cardiology Unit, Division of Internal Medicine, Faculty of Medicine, Prince of Songkla University, Hat Yai, Songkhla 90110, Thailand; Cardiology Unit, Division of Internal Medicine, Faculty of Medicine, Prince of Songkla University, Hat Yai, Songkhla 90110, Thailand; Translational Medicine Research Center, Faculty of Medicine, Prince of Songkla University, Hat Yai, Songkhla 90110, Thailand; Division of Surgery, Faculty of Medicine, Prince of Songkla University, Hat Yai, Songkhla 90110, Thailand; Endocrinology and Metabolism Unit, Division of Internal Medicine, Faculty of Medicine, Prince of Songkla University, Hat Yai, Songkhla 90110, Thailand

**Keywords:** primary aldosteronism, *KCNH2* mutation, long QT syndrome type 2, torsades de pointes, ventricular arrhythmia

## Abstract

Primary aldosteronism (PA) typically presents with hypertension and hypokalemia; however, its presentation as life-threatening ventricular arrhythmias is rare and often underrecognized. We report a case of a 30-year-old woman who presented with polymorphic ventricular tachycardia manifesting as torsades de pointes. Laboratory findings revealed persistent hypokalemia, metabolic alkalosis, suppressed renin, and inappropriately elevated aldosterone. Because recurrent malignant arrhythmia conferred a high immediate risk of sudden cardiac death, genetic testing for inherited arrhythmia syndromes was initiated, and an implantable cardioverter-defibrillator was implanted for secondary prevention before genetic confirmation became available. Endocrine evaluation proceeded concurrently and ultimately established the diagnosis of PA. Subsequent genetic analysis identified a pathogenic splice-site variant in *KCNH2*, consistent with congenital long QT syndrome type 2 (LQTS2). A review of the literature identified 16 previously reported cases of PA-associated ventricular arrhythmia, which showed a consistent association with hypokalemia and hypertension; however, none included genetic evaluation. This case highlights the need for parallel endocrine and arrhythmia evaluation in patients with unexplained ventricular arrhythmias.

## Introduction

Primary aldosteronism (PA) is a leading secondary cause of hypertension, accounting for 4% to 19% of hypertension cases across different clinical settings [1, 2]. Characterized by autonomous aldosterone secretion, PA results in volume expansion, hypokalemia, and metabolic alkalosis, which contribute to long-term cardiovascular and renal complications [3]. While cardiovascular complications of PA are well-recognized, its presentation with life-threatening arrhythmias such as torsades de pointes (TdP) remains exceedingly rare and often underrecognized. This report describes a young woman with recurrent ventricular arrhythmias ultimately diagnosed with both PA and a pathogenic splice-site variant in *KCNH2* consistent with congenital long QT syndrome type 2 (LQTS2), highlighting a novel intersection between endocrine disease and inherited cardiac channelopathy.

## Case presentation

A 30-year-old woman was admitted following a syncopal episode attributed to polymorphic ventricular tachycardia. Continuous cardiac monitoring revealed TdP in the setting of QT interval prolongation and hypokalemia. This was her first documented episode of sustained ventricular arrhythmia. She reported a 1-year history of intermittent palpitations without syncope, which had progressed over the preceding 3 months to monthly syncopal episodes, typically preceded by palpitations. She had no prior diagnoses of hypertension or other chronic illnesses. Slightly elevated blood pressure had been noted during a routine health screening, but was not pursued. There was no history of substance use, herbal supplements, or familial cardiovascular disease or sudden death.

On admission, her blood pressure was 130/80 mmHg with a regular pulse rate of 95 beats per minute, with symmetrical blood pressures and pulses in all extremities. The point of maximal impulse was at the 5th intercostal space, just lateral to the midclavicular line. Cardiac auscultation revealed no murmurs, and no abdominal bruits were detected. There were no clinical features suggestive of Cushing syndrome or acromegaly.

**Figure 1:**
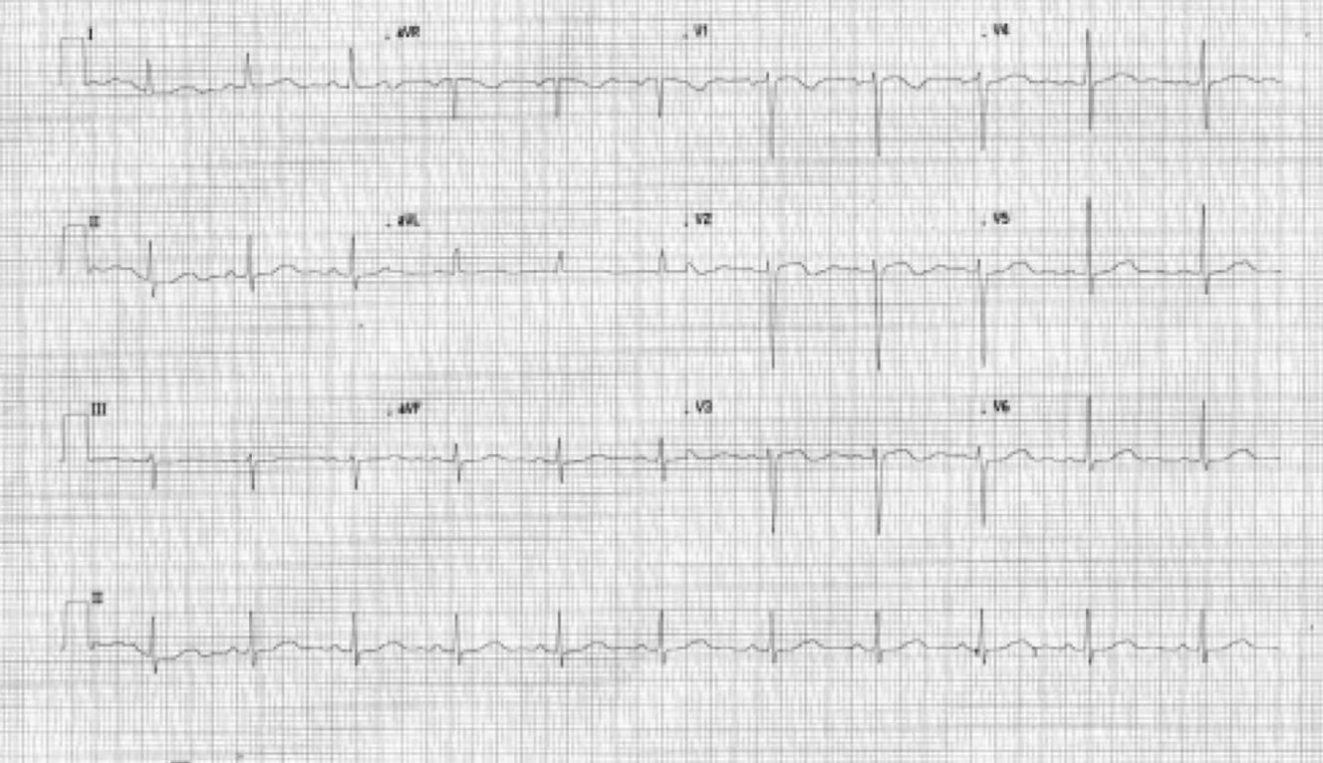
Initial electrocardiogram showing sinus rhythm with prominent U waves and prolonged QT interval.

Electrocardiography showed sinus rhythm with prominent U waves and a prolonged corrected QT interval (QTc) (Fig. 1). Chest radiography demonstrated mild cardiomegaly. Initial laboratory evaluation revealed significant hypokalemia, with a serum potassium concentration of 2.6 mmol/L (2.6 mEq/L) (reference range, 3.5-5.0 mmol/L [3.5-5.0 mEq/L]).

**Figure 2:**
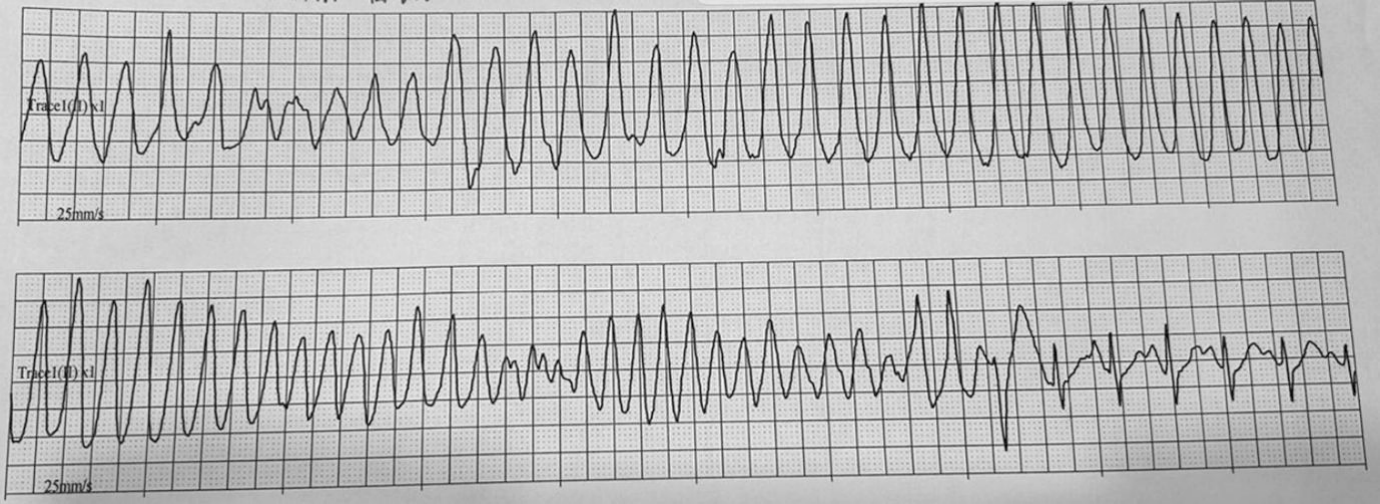
Continuous electrocardiographic monitoring showing polymorphic ventricular tachycardia consistent with torsades de pointes.

Bicarbonate was 25 mmol/L (25 mEq/L) (reference range, 22-29 mmol/L [22-29 mEq/L]). Sodium was 142 mmol/L (142 mEq/L) (reference range, 135-145 mmol/L [135-145 mEq/L]). Magnesium was 0.88 mmol/L (2.15 mg/dL) (reference range, 0.70-0.90 mmol/L [1.7-2.2 mg/dL]). Serum creatinine was 61 µmol/L (0.69 mg/dL) (reference range, 53-106 µmol/L [0.6-1.2 mg/dL]). Continuous cardiac monitoring captured recurrent episodes of polymorphic ventricular tachycardia, consistent with TdP (Fig. 2). A transthoracic echocardiogram revealed concentric left ventricular hypertrophy, suggestive of hypertensive heart disease.

Following potassium replacement, QTc remained prolonged at 524 ms with serum potassium normalized to 4.07 mmol/L (4.07 mEq/L). Serum magnesium was 0.89 mmol/L (2.17 mg/dL). No QT-prolonging medications were administered, aside from propranolol and potassium chloride. Because both arrhythmic and endocrine etiologies were suspected, cardiology and endocrine investigations were initiated concurrently. In the setting of persistent hypokalemia, metabolic alkalosis, and absence of diuretic use, a mineralocorticoid excess state was strongly suspected.

## Diagnostic assessment

Because the QT interval remained markedly prolonged despite normalization of serum potassium, an inherited arrhythmia syndrome was suspected, and genetic testing was initiated. However, the genetic result was not immediately available. Given recurrent TdP and the high near-term risk of sudden cardiac death, an implantable cardioverter-defibrillator (ICD) was implanted for secondary prevention before genetic confirmation was obtained. Subsequent genetic analysis identified a heterozygous pathogenic splice-site variant in *KCNH2* (c.2592+1G>A), consistent with LQTS2. During intensive care unit monitoring, the patient was noted to have repeated systolic blood pressure measurements in the 120 to 140 mmHg range. In conjunction with hypokalemia, metabolic alkalosis, and left ventricular hypertrophy, this finding raised clinical suspicion of a mineralocorticoid excess state, particularly PA. The plasma aldosterone concentration (PAC) was 427 pmol/L (15.46 ng/dL) (reference range 111-582 pmol/L [4.0-21.0 ng/dL]), and plasma renin activity (PRA) was markedly suppressed at 0.02 ng/mL/h (reference range 0.6-4.3 ng/mL/h), resulting in an aldosterone-to-renin ratio (ARR) of 773. The potassium level at the time was 4.51 mmol/L (4.51 mEq/L). Cushing syndrome and pheochromocytoma were excluded. At the time of renin and aldosterone measurement, the patient was taking propranolol. Because beta-blockade can suppress renin and artificially elevate the ARR, confirmatory testing was performed after propranolol withdrawal.

**Figure 3:**
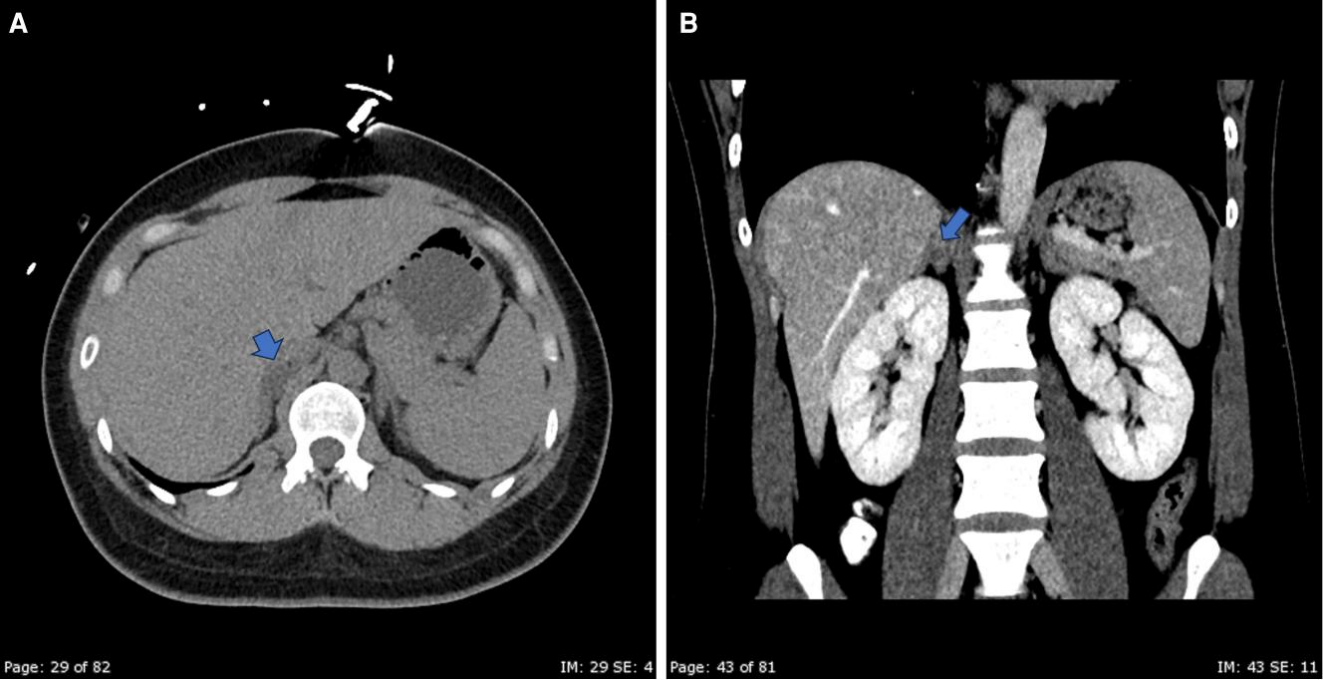
Axial (A) noncontrast and coronal (B) contrast-enhanced computed tomography (CT) images showing a well-defined, homogeneous right adrenal nodule (arrows), measured 1.3 cm, with unenhanced attenuation of 15 Hounsfield units. The coronal image highlights the spatial relationship of the lesion to the adjacent kidney and vasculature.

After withdrawal of propranolol, a saline infusion test confirmed autonomous aldosterone secretion. At 4 hours, the PAC remained elevated at 876 pmol/L (31.7 ng/dL), with a potassium level of 4.10 mmol/L (4.10 mEq/L). Spironolactone treatment was then initiated. Adrenal computed tomography (CT) identified a 1.3-cm homogeneous right adrenal nodule with an unenhanced attenuation of 15 Hounsfield units (HU) (Fig. 3). The left adrenal gland showed subtle prominence and diffuse thickening, without a focal lesion. Additional evaluation with adrenocorticotropic hormone (ACTH)-stimulated AVS was performed in this case as an individualized approach that extended beyond standard guideline recommendations [4]. This decision reflected the overall clinical context, including the presence of an underlying pathogenic genetic channelopathy and the subtle but atypical contralateral adrenal morphology. These factors raised concern for possible bilateral aldosterone secretion that could not be confidently excluded based on imaging alone. The test, as shown in Table 1, demonstrated suboptimal catheterization of the right adrenal vein. Right adrenal cortisol was 999 nmol/L (36.2 µg/dL) and inferior vena cava cortisol 778 nmol/L (28.2 µg/dL), resulting in a selectivity index of 1.3. In contrast, successful cannulation of the left adrenal vein was achieved, with cortisol 20 748 nmol/L (752 µg/dL) and a selectivity index of 26.6. The left aldosterone-to-cortisol (A/C) ratio was significantly suppressed at 0.14. The contralateral suppression index, calculated as the A/C ratio of the nondominant vein divided by the A/C ratio of the inferior vena cava, was 0.11, indicating contralateral suppression [5]. The serum potassium level at the time of AVS was 3.27 mmol/L (3.27 mEq/L). Together with concordant imaging findings, these results supported right-sided aldosterone excess.

**Table 1:** Adrenal venous sampling results supporting right-sided aldosterone excess

Parameter	Right adrenal vein	Inferior vena cava	Left adrenal vein	Reference range
Aldosterone	1115 pmol/L (40.2 ng/dL)	987 pmol/L (35.57 ng/dL)	2904 pmol/L (104.66 ng/dL)	111-582 pmol/L (4.00-21.00 ng/dL)
Cortisol	999 nmol/L (36.2 µg/dL)	778 nmol/L (28.2 µg/dL)	20 748 nmol/L (752 µg/dL)	138-690 nmol/L (5.0-25.0 µg/dL)
A/C ratio	1.11	1.26	0.14	—

Reference ranges apply to peripheral venous samples. AVS interpretation was based on selectivity indices and aldosterone-to-cortisol ratios, including contralateral suppression.

Abbreviations: A, aldosterone; C, cortisol; IVC, inferior vena cava.

## Treatment

**Figure 4:**
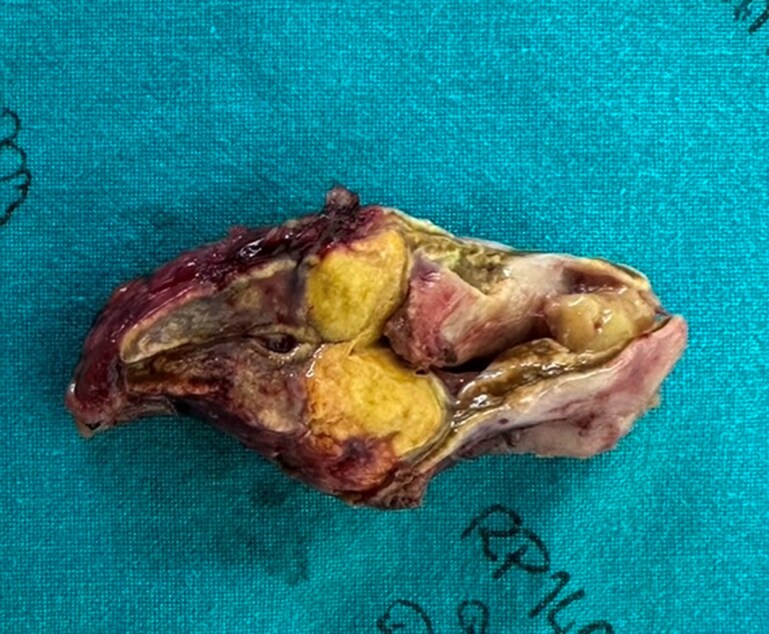
Gross photograph showing a well-circumscribed, yellow adrenal cortical adenoma.

**Figure 5:**
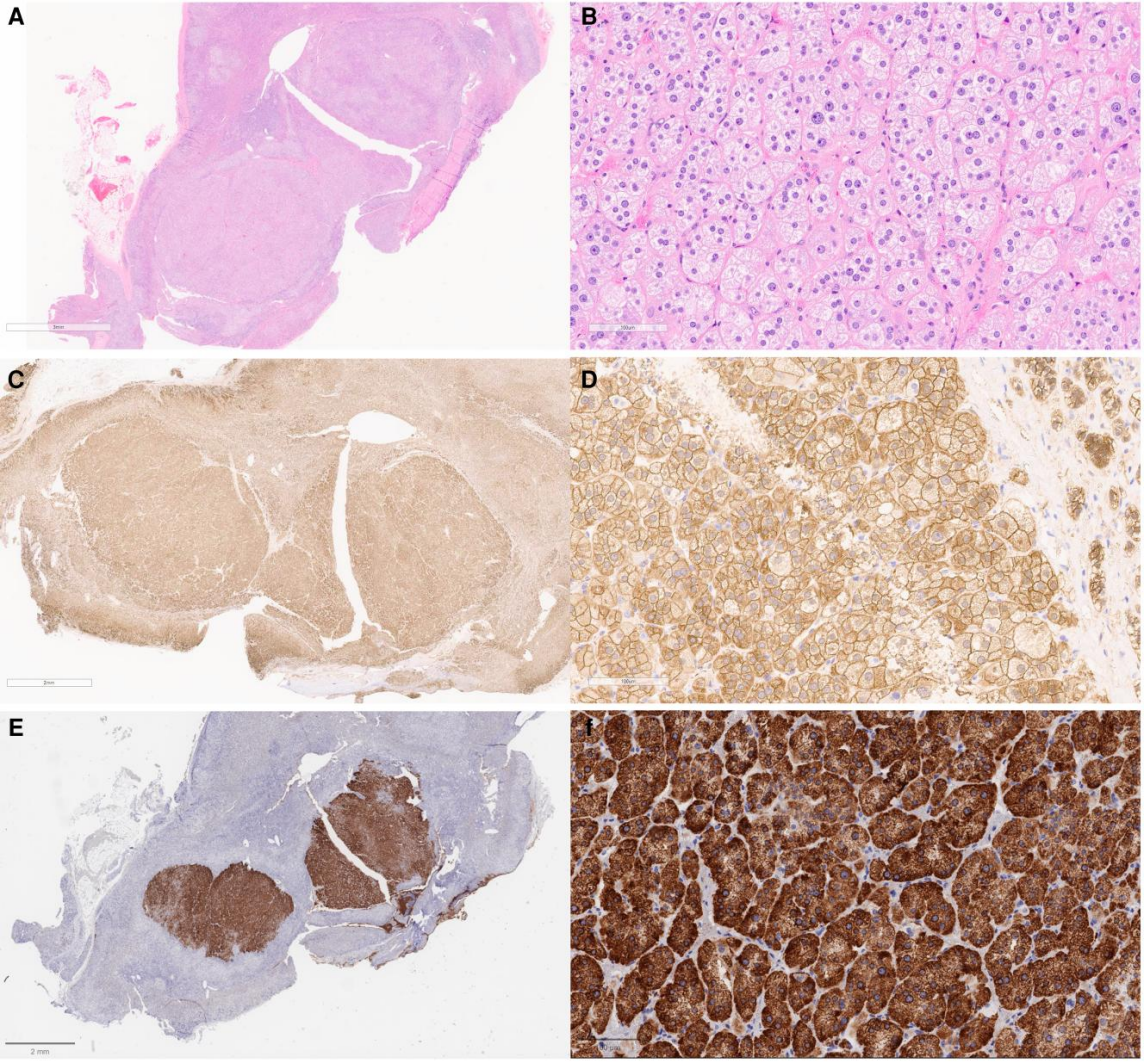
Histology and immunohistochemistry of the adrenocortical adenoma. (A) Low-power view showing a circumscribed 7-mm nodule located centrally within the adrenal cortex (H&E, ×20). (B) High-power view showing large lipid-rich tumor cells with abundant foamy cytoplasm, mild variation in nuclear size, and arrangement in cords and nests (H&E, ×200). (C, D) β-catenin immunostaining showing membranous staining without nuclear accumulation in the adenoma and adjacent adrenal tissue (β-catenin, ×20). (E, F) CYP11B2 immunostaining showing cytoplasmic positivity restricted to the adenoma (CYP11B2, ×20 and ×200, respectively).

The patient was well controlled preoperatively on spironolactone 100 mg daily and subsequently underwent laparoscopic right adrenalectomy. Gross examination revealed a well-circumscribed yellow adrenal cortical nodule measuring 1.0 × 1.0 × 1.0 cm^3^ (Fig. 4). Histopathologic evaluation confirmed a 0.7-cm adrenocortical adenoma. On hematoxylin and eosin staining, low-power examination showed a circumscribed cortical nodule, while high-power examination demonstrated large lipid-rich tumor cells with abundant foamy cytoplasm, mild nuclear size variation, and arrangement in cords and nests (Fig. 5A and B). Immunohistochemistry showed membranous β-catenin staining without nuclear accumulation in both the adenoma and the adjacent adrenal tissue (Fig. 5C and D). CYP11B2 immunostaining demonstrated cytoplasmic positivity restricted to the adenoma, confirming an aldosterone-producing adrenocortical adenoma (Fig. 5E and F).

## Outcome and follow-up

On postoperative day 1, PAC was 112 pmol/L (4.04 ng/dL) (reference range, 111-582 pmol/L [4.0-21.0 ng/dL]), with normalization of serum potassium to 4.12 mmol/L (reference range, 3.5-5.0 mmol/L). Following adrenalectomy, the patient remained normotensive and normokalemic without any medication. No further palpitations or syncopal episodes occurred during follow-up. At 6-month follow-up, serum potassium was 4.45 mmol/L (4.45 mEq/L), and bicarbonate was 20.7 mmol/L (20.7 mEq/L), with sustained arrhythmia-free status.

## Discussion

This case illustrates a rare but clinically important intersection between endocrine dysregulation and inherited cardiac channelopathy as a cause of life-threatening ventricular arrhythmia. Although atrial fibrillation is the most frequently reported arrhythmic manifestation in PA [6, 7], malignant ventricular arrhythmias such as TdP are uncommon and often underrecognized [8-11]. To better contextualize this presentation, we conducted a systematic literature review using PubMed/MEDLINE from inception to April 2025, applying the search terms: [“primary hyperaldosteronism” or “aldosterone-producing adenoma”] and [“ventricular arrhythmia” or “ventricular fibrillation”].

We identified 16 published cases of PA presentation with ventricular arrhythmia (Table 2). Among these, 75% were female, with a median age of 45 years (range, 26-78). Hypokalemia was universally reported; 62.5% had serum potassium <2.5 mmol/L (<2.5 mEq/L) and 43.8% had levels <2.0 mmol/L (<2.0 mEq/L). Hypertension was reported in 81.3%. Three patients with coexisting arrhythmic disorders underwent ICD placement [11, 15], while 2 additional patients received an ICD placement before PA was diagnosed [17]. Screening with PAC and PRA was performed in most cases, and adrenal adenomas were the most common subtype. Adrenalectomy was performed in 62.5% of patients and was generally associated with arrhythmia. However, none of the previously reported cases included genetic testing for inherited arrhythmia syndromes.

**Table 2:** Summary of reported cases of ventricular arrhythmia as the initial manifestation of primary aldosteronism

First author, year	Country	Age (yr)	Sex	Duration of HTN	Initial BP (mmHg)	Arrhythmia type	Serum potassium (mmol/L [mEq/L]; reference 3.5-5.0 mmol/L [3.5-5.0 mEq/L]	Plasma aldosterone concentration (pmol/L [ng/dL]; reference 100-400 pmol/L [3.6-14.4 ng/dL])	Plasma renin activity (ng/mL/h; reference 0.6-4.3 ng/mL/h)	ARR (reference <20)	Confirmatory test	AVS	Imaging	PA subtype	Treatment	Outcome/follow-up
Curry, 1976 [12]	USA	28	F	NA	160/100	Torsades de pointes, WPW	2.8 mmol/L [2.8 mEq/L]	24-hour urinary aldosterone 78.1 nmol/24 hours (28.2 µg/24 hours)	0.00	NA	SIT	Yes	Adrenal venography	Bilateral hyperplasia	Spironolactone, mexiletine	Arrhythmia controlled with medical therapy
Geist, 1996 [11]	Germany	45	F	23 years	NA	Torsades de pointes	2.0 mmol/L [2.0 mEq/L]	NA	NA	NA	NA	NA	CT	Adenoma	Adrenalectomy	Arrhythmia resolved
Geist, 1996 [11]	Germany	37	M	NA	NA	VT	2.5 mmol/L [2.5 mEq/L]	Elevated; exact value not reported	NA	NA	NA	NA	CT	Adenoma	Spironolactone	Improved arrhythmia control after treatment
Abdo, 1999 [13]	USA	37	F	NA	NA	VF	1.4 mmol/L [1.4 mEq/L]	1523.2 pmol/L (54.91 ng/dL)	0.40 ng/mL/h	137.28	NA	NA	NA	Adenoma	NA	NA
Sade, 2002 [10]	Israel	37	F	6 years	170/100	Torsades de pointes	2.1 mmol/L [2.1 mEq/L]	865.5 pmol/L (31.2 ng/dL)	Suppressed; exact value not reported	—	NA	NA	CT	Adenoma	Adrenalectomy	Arrhythmia resolved after adrenalectomy
Delgado, 2006 [14]	Spain	50	F	Yes	160/90	VF	1.6 mmol/L [mEq/L]	557.6 pmol/L (20.1 g/dL)	0.15 ng/mL/h	133.00	NA	NA	CT	Adenoma	Adrenalectomy planned	Improved with therapy
Furukawa,2007 [15]	Japan	60	F	16 years	NA	VF	1.8 mmol/L [1.8 mEq/L]	718.5 pmol/L (25.9 ng/dL)	0.30 ng/mL/h	86.33	NA	NA	NA	Adenoma	NA	NA
Shimony, 2009 [16]	Israel	31	M	NA	200/130	VF	2.3 mmol/L [2.3 mEq/L]	1328.8 pmol/L (47.9 ng/dL)	0.61 ng/mL/h	77.70	NA	NA	MRI	Carcinoma	Adrenalectomy	Rhythm normalized
Zelinka, 2009 [17]	Czech Republic	58	F	2 years	NA	Torsades de pointes	1.8 mmol/L [1.8 mEq/L]	651.0 pmol/L (23.5 ng/dL)	0.60 ng/mL/h	39.17	NA	NA	CT	Adenoma	Adrenalectomy	Full recovery
Zelinka, 2009 [17]	Czech Republic	42	F	Yes	NA	VT	2.5 mmol/L [2.5 mEq/L]	1464.7 pmol/L (52.8 ng/dL)	0.68 ng/mL/h	77.65	NA	NA	CT	Adenoma	Adrenalectomy	Partial improvement after adrenalectomy
Zelinka, 2009 [17]	Czech Republic	53	M	Yes	NA	Polymorphic VT, VF	3.1 mmol/L [3.1 mEq/L]	2535.4 pmol/L (91.4 ng/dL)	0.28 ng/mL/h	326.43	NA	NA	CT	Negative	Adrenalectomy	Arrhythmia resolved
Kornelius, 2012 [9]	Taiwan	78	M	10 years	138/78	Torsades de pointes	1.9 mmol/L [1.9 mEq/L]	1067.9 pmol/L (38.5 ng/dL)	0.67 ng/mL/h	57.72	NA	NA	CT	Adenoma	Adrenalectomy	Recovered postsurgery
Takamiya, 2014 [18]	Japan	45	F	1 year	NA	VF	2.2 mmol/L [2.2 mEq/L]	715.7 pmol/L (25.8 ng/dL)	<0.10 ng/mL/h	>258.00	CCT	Yes	CT	Adenoma	Adrenalectomy	Symptom resolution
Shao, 2018 [8]	China	74	M	20 years	153/90	VF	3.4 mmol/L [3.4 mEq/L]	832.2 pmol/L (30.0 ng/dL)	0.99 ng/mL/h	30.30	NA	NA	CT	Adenoma	Mineralocorticoid receptor antagonist	Improved BP and ECG
Costa Filho, 2023 [19]	Brazil	36	F	6 years	130/80	VF	1.0 mmol/L [1.0 mEq/L]	1251.1 pmol/L (45.1 ng/dL)	0.84 ng/mL/h	53.69	NA	NA	CT	Adenoma	Adrenalectomy	Normal ECG post-op
Hirose, 2024 [20]	Japan	26	F	NA	207/144	VF	2.0 mmol/L [2.0 mEq/L]	822.5 pmol/L (29.65 ng/dL)	0.20 ng/mL/h	148.25	CCT	Yes	CT	Adenoma	Adrenalectomy	Normalized BP and rhythm

For Curry et al [12], aldosterone was reported as 24-hour urinary aldosterone: 78.1 nmol/24 hours (28.2 µg/24 hours); reference range 2.8-44.3 nmol/24 hours (1-16 µg/24 hours). ARR reference threshold based on commonly used screening criteria (PAC >15 ng/dL with suppressed renin).

Abbreviations: ARR, aldosterone–renin ratio; AVS, adrenal vein sampling; BP, blood pressure; CCT, captopril challenge test; CT, computed tomography; ECG, electrocardiogram; F, female; HTN, hypertension; M, male; MRI, magnetic resonance imaging; NA, not available; PA, primary aldosteronism; SIT, saline infusion test; VF, ventricular fibrillation; VT, ventricular tachycardia; WPW, Wolff–Parkinson–White syndrome.

To our knowledge, this is the first reported case of PA presenting with malignant ventricular arrhythmia in which a pathogenic *KCNH2* variant causing LQTS2 was identified. The persistence of QT prolongation despite normalization of serum potassium raised suspicion for an underlying inherited arrhythmia syndrome and prompted genetic evaluation. Recognition of both conditions enabled a precision-based management strategy combining ICD placement and targeted treatment of mineralocorticoid excess.

Histopathologic evaluation further supported the diagnosis of an aldosterone-producing tumor. The tumor demonstrated typical morphologic features of adrenocortical adenoma with lipid-rich cells arranged in cords and nests. CYP11B2 immunostaining confirmed aldosterone production localized to the adenoma, supporting unilateral PA and concordance with the biochemical and AVS findings.

*KCNH2* encodes the α-subunit of the rapid delayed rectifier potassium current (IKr), which is critical for cardiac repolarization. Variants in *KCNH2* reduce IKr current, impair repolarization reserve, and prolong the QT interval, predisposing to TdP. Pathogenic variants in *KCNH2* account for approximately 30% to 45% of inherited LQTS cases [21]. While the cardiac role of *KCNH2* is well understood, its expression in adrenal tissue raises the possibility of involvement in aldosterone synthesis, a hypothesis yet to be validated clinically.

Aldosterone synthesis in the adrenal cortex is regulated by calcium signaling pathways activated by potassium and angiotensin II. Pathogenic variants in ion channels and pumps such as *KCNJ5*, *CACNA1D*, *CLCN2*, *ATP1A1*, and *ATP2B3* promote calcium influx and aldosterone overproduction [22-25]. *KCNJ5* is the most frequently mutated gene, and its mutations are more prevalent among Asian populations [26]. Recent reports have also documented somatic mutations in *CACNA1H*, *CADM1*, *SLC30A1*, and *PRKACA*; however, these mutations are significantly less frequent [26].

Expression of *KCNH2* in adrenal tissue has been documented [27]. Whether dysfunction of *KCNH2* contributes to dysregulated aldosterone synthesis remains speculative; however, the coexistence of these conditions raises the possibility of shared channelopathy mechanisms affecting both cardiac repolarization and adrenal steroidogenesis.

LQTS may also be acquired, most commonly due to medications, electrolyte imbalance, cardiomyopathies, myocardial infarction, or increased intracranial pressure [28]. Inherited LQTS is usually transmitted in an autosomal dominant pattern, with 5% to 10% arising from *de novo* mutations [29].

This case highlights the importance of integrated cardiovascular and endocrine evaluation in patients presenting with malignant ventricular arrhythmias and unexplained hypokalemia, and suggests that germline channelopathies may have broader systemic implications than previously recognized.

## Learning points

- Primary aldosteronism (PA) may present with life-threatening ventricular arrhythmias, particularly in the setting of severe hypokalemia.
- Persistent QTc prolongation despite correction of electrolyte abnormalities should prompt evaluation for an inherited arrhythmic syndrome.
- Genetic testing may reveal coexisting channelopathies that influence long-term risk stratification and management.
- Adrenal vein sampling remains essential for subtype classification of PA when imaging findings are inconclusive.
- Germline ion-channel variants such as *KCNH2* may have broader systemic implications beyond cardiac electrophysiology.

## Data Availability

Data sharing is not applicable to this article as no datasets were generated or analyzed during the current study.
